# Epithelialization of mouse ovarian tumor cells originating in the fallopian tube stroma

**DOI:** 10.18632/oncotarget.11808

**Published:** 2016-09-01

**Authors:** Yuanyuan Hua, Pui-Wah Choi, Alexander J. Trachtenberg, Allen C. Ng, Winston P. Kuo, Shu-Kay Ng, Daniela M. Dinulescu, Martin M. Matzuk, Ross S. Berkowitz, Shu-Wing Ng

**Affiliations:** ^1^ Department of Obstetrics & Gynecology, The Second Affiliated Hospital of Chongqing Medical University, Chongqing, PR China; ^2^ Department of Obstetrics/Gynecology and Reproductive Biology, Brigham and Women's Hospital, Boston, Massachusetts, USA; ^3^ Harvard Catalyst Laboratory for Innovative Translational Technologies, Harvard Medical School, Boston, Massachusetts, USA; ^4^ Predicine, Inc., Hayward, California, USA; ^5^ School of Medicine and Menzies Health Institute Queensland, Griffith University, Nathan, Australia; ^6^ Department of Pathology, Brigham and Women's Hospital, Boston, Massachusetts, USA; ^7^ Dan L. Duncan Cancer Center, Baylor College of Medicine, Houston, Texas, USA

**Keywords:** ovarian cancer, fallopian tube

## Abstract

Epithelial ovarian carcinoma accounts for 90% of all ovarian cancer and is the most deadly gynecologic malignancy. Recent studies have suggested that fallopian tube fimbriae can be the origin of cells for high-grade serous subtype of epithelial ovarian carcinoma (HGSOC). A mouse HGSOC model with conditional *Dicer-Pten* double knockout (*Dicer-Pten* DKO) developed primary tumors, intriguingly, from the fallopian tube stroma. We examined the growth and epithelial phenotypes of the *Dicer-Pten* DKO mouse tumor cells contributable by each gene knockout. Unlike human ovarian epithelial cancer cells that expressed full-length E-cadherin, the *Dicer-Pten* DKO stromal tumor cells expressed cleaved E-cadherin fragments and metalloproteinase 2, a mixture of epithelial and mesenchymal markers. Although the *Dicer-Pten* DKO tumor cells lost the expression of mature microRNAs as expected, they showed high levels of tRNA fragment expression and enhanced AKT activation due to the loss of PTEN function. Introduction of a *Dicer1-*expressing construct into the DKO mouse tumor cells significantly reduced DNA synthesis and the cell growth rate, with concurrent diminished adhesion and ZO1 epithelial staining. Hence, it is likely that the loss of *Dicer* promoted mesenchymal-epithelial transition in fallopian tube stromal cells, and in conjunction with *Pten* loss, further promoted cell proliferation and epithelial-like tumorigenesis.

## INTRODUCTION

Epithelial ovarian cancer is the deadliest of all gynecologic malignancies and is the fifth leading cause of cancer death in females in the United States [1]. Because the lack of a highly specific early detection marker and frequent relapses, the majority of ovarian cancer patients are usually diagnosed at an advanced stage and have a 5-year survival rate of about 30% [1]. High-grade serous ovarian carcinoma (HGSOC) is the most common and aggressive type of ovarian neoplasm with epithelial cells resembling those of fallopian tube, comprising about 50% of primary epithelial ovarian tumors [2]. Historically, ovarian surface epithelial (OSE) cells on the ovarian surface epithelium and the associated cortical inclusion cysts have been suggested as the cells of origin for ovarian cancer [3, 4]. Recent studies, however, have suggested that epithelial cells in the distal fallopian tube can be the cells of origin for the development of HGSOC. Serous tubal intraepithelial carcinomas (STIC) with both *p53* mutations and expression of γ-H2AX, evidence of DNA damage that is frequently observed in HGSOC, are proposed as a potential precursor for HGSOC. [5–8].

Several mouse models with genomic manipulations in specific organ sites have been established for ovarian tumors originating from ovarian surface epithelia [9–12] and fallopian tube [13], respectively. Mechanistic studies of these mouse models may provide insights into the mechanisms by which native human ovarian cancer develops and is regulated. One recent mouse model employed anti-Muüllerian hormone receptor type 2-directed Cre (*Amhr2-Cre*) to specifically knockout both *Dicer* and *Pten* genes in the mouse female reproductive tract [14]. The *Dicer-Pten* DKO (*Dicer^flox/flox^*Pten*^flox/flox^*Amhr2*^cre/+^*) mice developed early serous carcinomas in the fallopian tube, which mimicked the morphology and clinical manifestation in human HGSOC. However, intriguingly, the primary epithelial tumors were found to arise from cells of a mesenchymal lineage in the stroma of the fallopian tube [14]. The contribution of *Pten* dysregulation in ovarian cancer has been well researched in human ovarian cancer and mouse models [9, 10, 15–17], and the tumors arose from epithelial cells in the mouse models. But for *Dicer1*, germline mutations have been found in familial pleuropulmonary blastoma, a rare pediatric lung cancer [18], embryonal rhabdomyosarcomas [19], and familial multinodular goiter [20]. Interestingly, somatic *Dicer1* hotspot missense mutations with defective *Dicer1* function in 5p miRNA production were commonly found in nonepithelial ovarian tumors, in particular in 60% of Sertoli-Leydig cell tumors, and rarely in epithelial ovarian and endometrial carcinomas [21, 22]. Given the predominance of *Dicer1* mutations in nonepithelial ovarian tumors, the appearance of epithelial HGSOC tumors arising from the fallopian tube stroma in the *Dicer-Pten* DKO mouse model might be likely due to the loss of *Dicer1* function.

Molecular characterization of ovarian tumors and cancer cell lines has shown that they are more epithelial-like than normal ovarian surface epithelia and the derived cell lines [3, 4, 23, 24], which possess both mesenchymal and epithelial characteristics for post-ovulatory wound healing and tissue homeostasis [3, 25]. The expression of adherens junction protein E-cadherin was elevated in ovarian tumors [26] and ectopic expression of E-cadherin in OSE caused mesenchymal-epithelial transition and the resulting cells formed tumors in immunodeficient mice [27, 28]. Our previous sequential *in vitro* three-dimensional culture models have also shown that E-cadherin function is important for ovarian inclusion cyst formation and ovarian tumor invasion [29]. In this study, we examined the epithelial phenotypes of the *Dicer-Pten* DKO mouse tumor cells and contribution of each knockout genes in tumor phenotypes.

## RESULTS

### Epithelial phenotypes of the *Dicer-Pten* DKO mouse tumors and cancer cell lines

**Figure 1:**
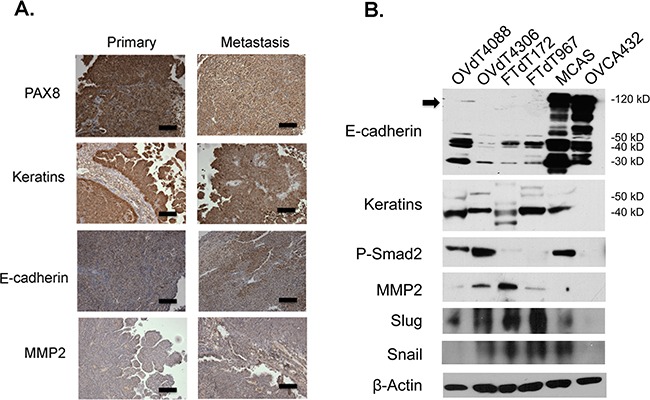
The *Dicer-Pten* DKO mouse tumor cells express a mixture of epithelial and mesenchymal markers **A.** Immunohistochemistry of the *Dicer-Pten* DKO mouse tumor tissues for different markers. Scale bars represent 50μm. **B.** Western blot analysis of marker expression in different cell lysates. The position of the full-length E-cadherin is marked by an arrowhead. β–actin was used as loading control.

We first investigated the epithelial phenotypes of the *Dicer-Pten* DKO mouse tumors by performing immunohistochemistry for the expression of epithelial and mesenchymal markers (Figure 1A). Both the primary and metastatic tumors stained positive for PAX8, a marker for embryonic Müllerian ducts, human fallopian tubes, and serous subtype of ovarian carcinomas [30]. The tumors also had high expression of cytokeratins. However, the tumors showed modest positive staining of adherens junction protein, E-cadherin, and matrix metalloproteinase-2 (MMP2) that are associated with epithelial-mesenchymal-transition (EMT). We also examined the epithelial phenotypes of the *Dicer-Pten* DKO fallopian tube tumor-derived cancer cell lines (FTdT172 and FTdT967) together with two mouse cancer cell lines originated from the ovarian surface epithelium, OVdT4306 and OVdT4088, which were derived from *K-ras*^G12D/+^*Pten*^−/−^ and *K-ras*^G12D/+^ *TP53*^−/−^ mice, respectively, [9]. We also employed two human ovarian cancer cell lines, OVCA432 and MCAS, in the comparison. Western blot analysis of the cell lysates (Figure 1B) showed that all cancer cell lines except OVCA432 expressed epithelial cytokeratins. The human ovarian cancer cell lines expressed high levels of full-length E-cadherin proteins, whereas the mouse ovarian tumor- and fallopian tube tumor-derived cancer cell lines expressed only E-cadherin protein fragments with smaller molecular weights. Furthermore, as reported before [29], the human ovarian cancer cells did not express MMP2, which was expressed in the mouse tumor cell lines. Within the mouse tumor cell lines, the ovarian tumor-derived OVdT4088 and OVdT4306 cancer cells expressed the TGFβ downstream target phosphorylated Smad2, while the *Dicer-Pten* DKO cancer cell lines showed very little expression. Instead, the *Dicer-Pten* DKO cancer cell lines had higher expression of TGFβ downstream transcription factors Slug and Snail. Hence, the expression analysis showed that the *Dicer-Pten* DKO mouse fallopian tube tumors and cancer cells expressed a mixture of epithelial and mesenchymal markers, that were very distinct from human epithelial ovarian cancer cells.

### Investigation of cell growth and small RNA expression phenotypes of the *Dicer-Pten* DKO mouse tumors and cancer cell lines

**Figure 2:**
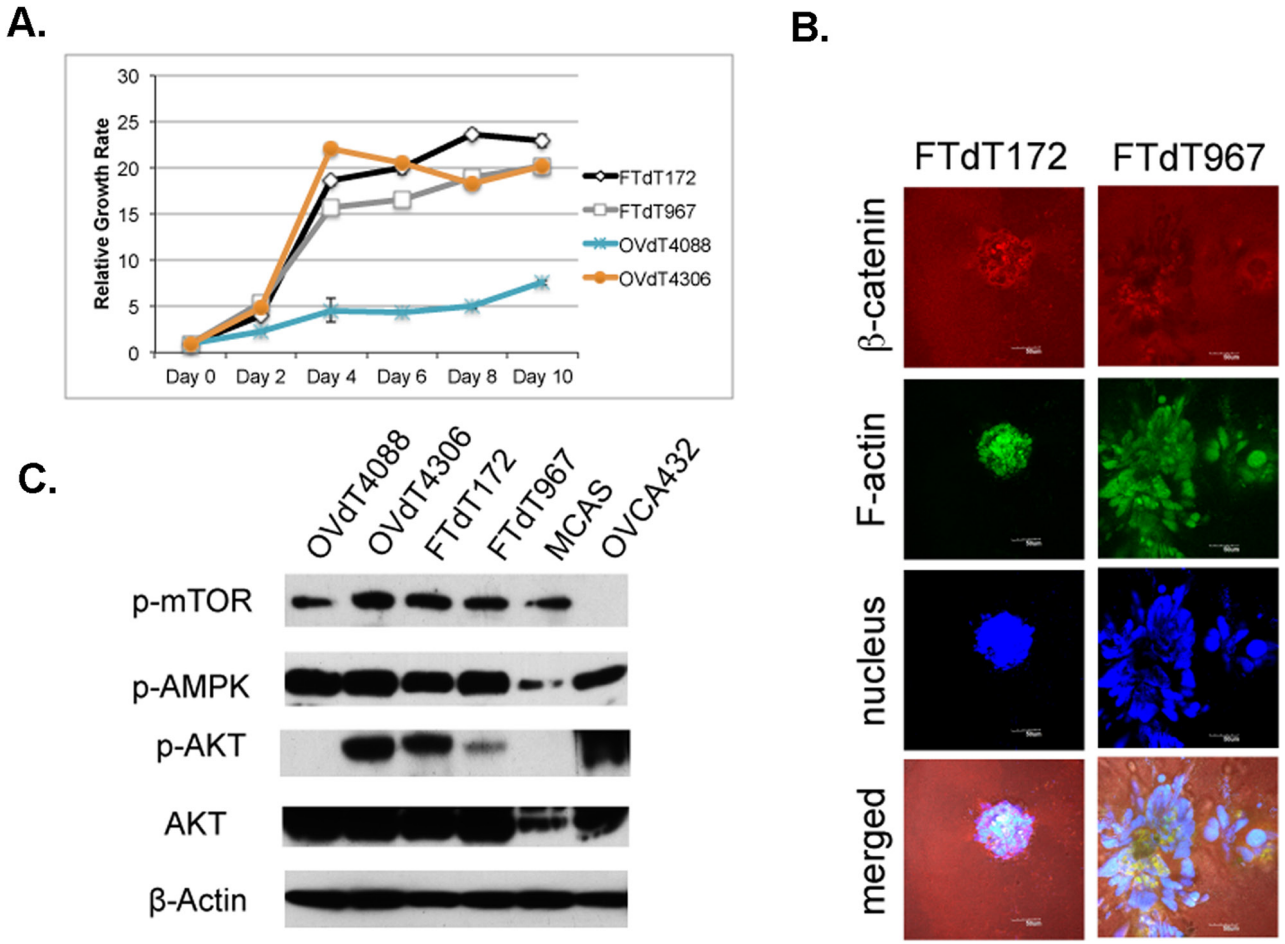
The *Pten* disruption contributed to the rapid proliferation of the *Dicer-Pten* DKO mouse cancer cells **A.** Cell growth assay to compare the growth rates of different mouse cancer cell lines. B. Confocal microscopic images of three-dimensional *in vitro* spheres formed by the *Dicer-Pten* DKO mouse cancer cell lines in Collagen I matrix and stained for β–catenin (red), F-actin (green) and nucleus (blue). **C.** Western blot analysis for the expression for growth and metabolic markers. β–actin was used as loading control.

As HGSOC is a highly aggressive tumor, we compared the growth rate among the mouse tumor cell lines (Figure 2A). Both *Dicer-Pten* DKO cancer cell lines and the OVdT4306 cancer line showed enhanced growth rate compared with the *K-ras*^G12D/+^ *TP53*^−/−^ OVdT4088 mouse cancer cell line. We also tested the two *Dicer-Pten* DKO cancer cell lines in a sequential three-dimensional culture system which we have previously developed [29]. The FTdT967 line showed more aggressive growth and invaded into the collagen I extracellular matrix after 3 days of growth (Figure 2B), suggesting that this line is derived from a tumor that may have a more aggressive phenotype. Both *Dicer-Pten* DKO tumor lines and the OVdT4306 cancer cell line shared genomic *Pten* deletion, which affected the PI3K/AKT key metabolic and cell growth pathway. We investigated the status of AKT pathway in different cancer cell lines by Western blot analysis (Figure 2C). These three mouse cancer cell lines showed increased phospho-AKT expression when compared with the OVdT4088 line, and expectedly, showed stronger expression of the downstream phospho-mTOR target. However, within these four mouse cell lines, there was no change in phospho-AMPK, another master regulator of cell growth and metabolism that can affect mTOR activity [31].

**Figure 3:**
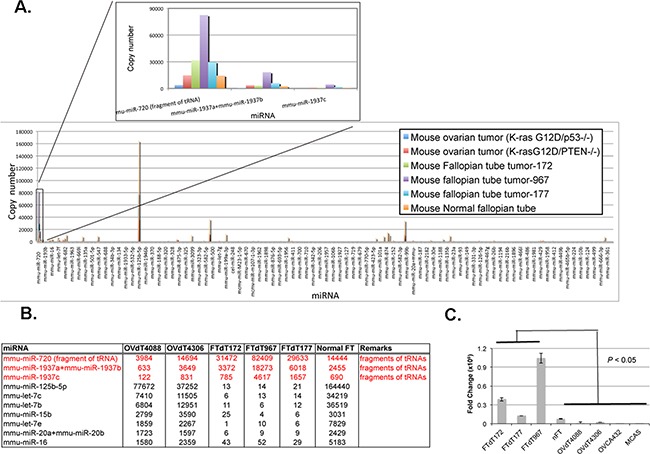
The *Dicer-Pten* DKO mouse cancer cells expressed elevated levels of tRNA fragments **A.** The result of the Nanostring® nCounter miRNA expression assay. The relative expression of mmu-miR-720, mmu-miR-1937a, 1937b, and 1937c in different cell lines is shown in the inset. **B.** Normalized counts of the miRNAs representing tRNA fragments that are elevated (red), as well as some other miRNAs that are significantly down-regulated (dark) in the *Dicer-Pten* DKO mouse cancer cells relative to normal fallopian tube (FT) and ovary-derived tumor cell lines. **C.** qRT-PCR of mmu-miR-720 in different RNA preparations.

As our preliminary qRT-PCR test showed some minor differences in the miRNA expression in the *Dicer-Pten* DKO cancer cells (data not shown), we carried out a miRNA transcriptomic profiling of the *Dicer-Pten* DKO tumor lines 172, 177, and 967. For comparison, we also determined the miRNA profiles from RNA isolated from normal mouse fallopian tubes, and the two ovary-derived lines. The results of the profiling (Figure 3A and Figure 3B) showed that while most of all the miRNA species examined showed downregulation in the *Dicer-Pten* DKO tumor lines, there were significantly elevated levels of miR-720 and miR-1937a, b, and c in the DKO tumor lines, which according to miRBase database (http://www.mirbase.org/index.shtml), belonged to tRNA fragments. qRT-PCR performed on the RNA preparations validated the elevated expression of miR-720 tRNA fragments in the three *Dicer-Pten* DKO tumor lines relative to normal fallopian tube and the other cancer cell lines (Figure 3C, *P* < 0.05). Hence, excessive tRNAs were expressed in these DKO lines despite the downregulation of miRNA expression.

### Introduction of a *Dicer1*-expression construct into the *Dicer-Pten* DKO mouse cancer cells reversed the growth and epithelial phenotypes

**Figure 4:**
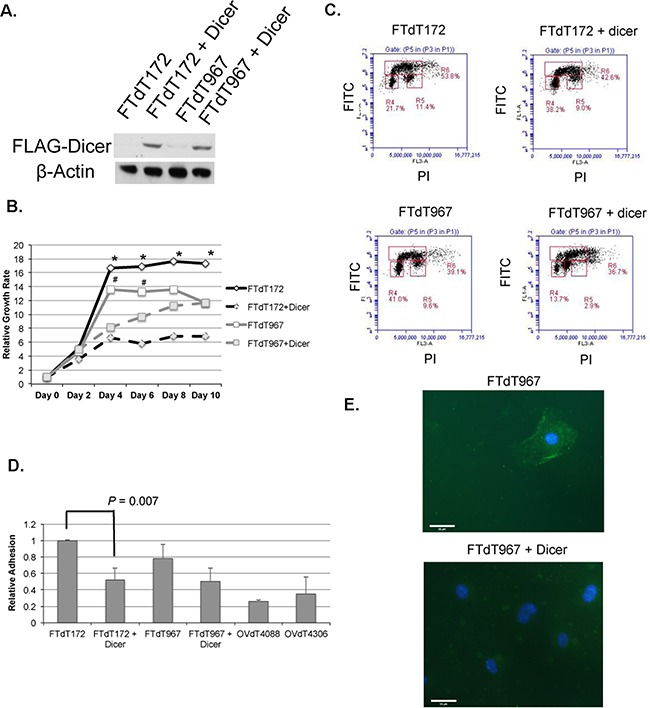
Introduction of a *Dicer1*-expression construct reversed the epithelial and growth phenotypes of the *Dicer-Pten* DKO mouse cancer cells **A.** Western blot to confirm the expression of FLAG-tagged Dicer1 in the lentivirus-infected cancer cells. β–actin was used as loading control. **B.** Cell growth assay to compare the growth rates of control and lentivirus-infected cancer cell lines. ^*^, *P* < 0.005; ^#^, *P* < 0.02. **C.** Flow cytometric density graphs to show the BrdU incorporation and cell cycles of the mouse cancer cell lines. The Y-axis represents the FITC fluorescence of BrdU labeled cells, whereas the X-axis represents the propidium iodide (PI) fluorescence. The boxed areas show the percentages of cell populations in G0/G1 phase (R4), G2/M phase (R5), and S phase (R6), respectively. **D.** Relative adhesion of different cell lines to collagen I extracellular matrix. The reading of FTdT172 cells was defined as 1. **E.** Fluorescence micrographs of ZO-1 staining (green) in both FTdT967 cells (top) and FTdT967 cells infected with *Dicer1*-expressing lentivirus (bottom). The DAPI-stained nuclei were shown in blue.

To investigate whether *Dicer1* knockout caused the epithelialization of the mouse fallopian tube stromal cells and affected the growth of the *Dicer-Pten* DKO cancer cells, a FLAG-tagged *Dicer1*-expression construct was introduced into both FTdT172 and FTdT967 cells by lentiviral infection and the resulting cells were tested for FLAG-Dicer1 expression (Figure 4A) and cell growth and epithelial phenotypes. Compared to control cells, *Dicer1*-lentivirus infected cells showed significant reduction in growth rate (Figure 4B). Cell cycle analysis of BrdU- and propidium iodide-labeled cells by flow cytometry also showed that the *Dicer1*-lentivirus infected cells had significant reduction in S and G2M phases in the cell cycle (Figure 4C). Intriguingly, *Dicer1*-lentivirus infected FTdT967 cells showed an additional subpopulation of cells between G1 and G2/M. An adhesion assay showed that the *Dicer1*-lentivirus infected FTdT172 cells showed significant reduced adherence to collagen I extracellular matrix than the control cells (Figure 4D, *P* = 0.007). The *Dicer1*-lentivirus infected FTdT967 cells also showed reduced, albeit not significant, cell adherence when compared with the control cells. Nevertheless, they demonstrated loss of membrane-bound expression of the tight junction-associated signaling protein ZO-1 when compared with control cells (Figure 4E).

## DISCUSSION

Recent studies have suggested that the epithelial cells in distal fallopian tube can be the cells of origin for the development of HGSOC [5–8]. A genetically engineered mouse model that disrupted *Brca1/Brca2, Tp53*, and *Pten* genes specifically in fallopian tube epithelial cells developed carcinoma that resembled HGSOC [13]. The fact that the tumor development was preventable with the removal of fallopian tube, but not with the removal of the ovaries supported the notion of fallopian tube origin of HGSOC. It is interesting to note that the *Dicer-Pten* DKO mouse model developed HGSOC-like tumors originating from the stroma of fallopian tube[14], as human HGSOC tumor cells are believed to be derived from epithelial cells that express many epithelial markers [23, 24]. We examined the tumor tissues and derived cancer cell lines and showed that the tumor cells expressed a mixture of epithelial and mesenchymal markers (Figure 1). Actually, the marker expression pattern of the *Dicer-Pten* DKO tumor cells was similar to that represented by the mouse model established from the mesothelial-like ovarian surface epithelial cells [9]. While both fallopian tube tumor- and ovarian tumor-derived mouse model cell lines expressed epithelial cytokeratins, they expressed only E-cadherin cleaved fragments and the EMT marker MMP2. The mouse cancer cell lines also expressed higher levels of TGFβ pathway downstream effector phosphorylated Smad2 or transcription factors Slug and Snail. Therefore, while these tumors established epithelial-like properties, they maintained certain mesenchymal characteristics that were distinct from human HGSOC cells. It is also true that the fast growing mouse tumor cells would detach as skin when confluent (data not shown), suggesting some mesenchymal nature of these mouse cancer cells was preserved.

Given the predominance of *Dicer1* mutations in nonepithelial ovarian tumors [21], it was expected that the knockout of the *Dicer1* gene was responsible for the epithelialization of the mouse mesenchymal cells to form epithelial tumors in the *Dicer-Pten* DKO mouse model. Indeed, introduction of a *Dicer1*-expressing construct to the *Dicer-Pten* DKO mouse cancer cells reversed some epithelial phenotypes such as cell adhesion (Figure 4D). Even though the more aggressive growing FTdT967 cells did not show significant reduction in cell adhesion after the reintroduction of *Dicer1*, the resulting cells nevertheless showed loss of ZO-1 membrane localization of the control cells (Figure 4E). ZO-1 is a tight junction-associated signaling protein that is important for epithelial cell differentiation [32], the cytoplasmic distribution of ZO-1 in the *Dicer1*-reintroduced FTdT967 cells suggested that these cells lost the epithelial phenotype as did the *Dicer1*-reintroduced FTdT172 cells. Hence, loss of *Dicer1* in the *Dicer-Pten* DKO mouse model likely contributed to the epithelialization of stromal cells. Specific knockouts of *Dicer1* in other mouse cell types have been reported to affect various cell differentiation and organ development. Disrupting *Dicer1* function specifically in thyrocytes has shown downregulation of thyrocyte cell adhesion proteins and cell differentiation, which led to severe hypothyroidism and shortened life span [33]. It is also of interest to note that loss of Dicer in epicardium-derived progenitor cells impaired EMT of the progenitor cells and a reduction in epicardial cell proliferation and differentiation into different cell lineages [34]. Hence, it is important to delineate the consequences of Dicer1 dysregulations in a cell context-dependent manner.

We next investigated the contribution of *Dicer* and *Pten* gene knockouts on cell growth. Since *Pten* is known for suppressing PI3K/AKT pathway for cell growth and cell metabolism, the effects of *Pten* knockout on the activation of AKT and fast cell growth are evident in our assays (Figure 2). Furthermore, our miRNA profiling experiment resulted in the identification of tRNA overexpression in the *Dicer-Pten* DKO mouse tumors relative to normal fallopian tube cells (Figure 3). PTEN has been reported to act through PI3K/AKT/mTOR pathway to suppress RNA polymerase III-mediated tRNA transcription [35, 36] and enhanced RNA polymerase III activity has been shown to promote oncogenic transformation [37, 38]. Individual tRNA fragments such as miR-720 have also been reported to promote cell migration [39] and represent a signature of breast cancer-derived extracellular vesicles [40]. Hence, it is likely that the loss of *Pten* in the *Dicer-Pten* DKO mouse model resulted in upregulation of RNA polymerase III transcription and the tRNA overexpression contributed to oncogenic transformation of the fallopian tube stromal cells.

Besides the contribution by *Pten* disruption, our studies showed that *Dicer1* disruption also contributed to the rapid cell proliferation of the DKO mouse cancer cells (Figure 4B and 4C). Our finding is consistent with the report by Chen et al. [22] that cancer cells harboring *Dicer1* hotspot mutants that had lost 5p miRNA biogenesis, showed increased proliferation rate and upregulated expression of cell cycle promoting genes compared to cancer cells with wild-type *Dicer1*, suggesting the tumor-suppressing effect of *Dicer1* in wild-type cells. Similar to the *Dicer1* hotspot mutant cells, the *Dicer-Pten* DKO mouse tumor cells showed significant reduced expression of miRNAs such as let-7 and miR-125 (Figure 3B), which have been shown to harbor tumor suppressor functions [22, 41].

In conclusion, our studies have delineated the contribution of the *Dicer* and *Pten* disruptions to the DKO tumor cells originated in fallopian tube stroma. The loss of *Pten* contributed to the activated AKT pathway and increased RNA polymerase III transcription, which led to rapid proliferation of the cancer cells. However, disruption of *Dicer1* played a dominant role in epithelialization of fallopian tube stromal cells, and in conjunction with *Pten* loss, drove these mesenchymal cells to rapid growth and epithelial-like tumorigenesis. *Dicer1* dysregulations in epithelial cancers like ovarian and endometrial carcinomas are not as common as in nonepithelial cancers. The results of Cancer Genome Atlas (TCGA) genomic analyses of HGSOC revealed that 2.5% of HGSOC had alterations of *Dicer1* gene [42]. Similar to the study of *Dicer1* hotspot mutations in endometrial cancer [43], further studies of the potential role of *Dicer1* dysregulations in affecting stromal cells in fallopian tube will provide insights into this potential route of ovarian cancer development.

## MATERIALS AND METHODS

### Cell lines and mouse tumor tissues

The *Dicer-Pten* DKO (*Dicer^flox/flox^*Pten*^flox/flox^*Amhr2*^cre/+^*) mouse tumors and cancer cell lines have been previously described [14]. As a reference, the two mouse ovarian cancer cell lines derived from *K-ras*^G12D/+^*Pten*^−/−^ and *K-ras*^G12D/+^ *TP53*^−/−^ mice, respectively, [9] were used. Ovarian cancer cell lines OVCA432 and MCAS have been described before [44]. The human ovarian cancer cell lines were grown in medium 199 (Sigma-Aldrich, St. Louis, MO) and MCDB 105 (Sigma-Aldrich, St. Louis, MO) (1:1) supplemented with 10% fetal calf serum (FCS). The mouse tumor cell lines were cultured with DMEM-F12 HAM medium (Life Technologies, Grand Island, NY) plus 10% FCS.

### Immunohistochemistry

Immunohistochemistry (IHC) was performed on archived formalin-fixed, paraffin-embedded *Dicer-Pten* DKO mouse tumors tissues. Standard xylene deparaffinization, rehydration with a descending series of ethanol solutions, antigen retrieval (Vector Laboratories, Burlingame, CA), and blocking of endogenous peroxidases in 0.3% H_2_O_2_ were performed as described before [44]. 3, 3 –diaminobenzidine (DAB) horseradish peroxidase substrate kit was used for color development (Vector Laboratories, Burlingame, CA).

### Cell growth assay, three-dimensional cultures, and Western blot analysis

Cell growth study was performed by seeding 5×10^3^ cells per well in 96-well microtiter plates and the growth was determined by measuring methylthiazol tetrazolium (MTT) (5 mg/mL in PBS, Sigma-Aldrich, Natick, MA) incorporation on different days. Absorbance at 562 nm was determined on an ELx800 absorbance microplate reader (Bio-Tek, Winooski, VT). All the growth assays were performed in triplicates and repeated twice.

For three-dimensional cultures, single mouse cancer cells were allowed to form spheroids in 2% Matrigel (BD Biosciences, San Jose, CA), and the spheroids were transferred to Collagen I extracellular matrix (Sigma-Aldrich, St. Louis, MO) for 3 days, fixed and stained with antibody for β-catenin (BD Biosciences, San Jose, CA) and phalloidin (Invitrogen, Carlsbad, CA) for F-actin, countered stained with Sytox Green (Invitrogen, Carlsbad, CA) as described before [29]. Confocal images were captured using a Leica SP5 confocal microscope (Leica Microsystems, Bannockburn, IL) and analyzed by the Leica LAS AF software (Leica Microsystems, Bannockburn, IL).

Total cell lysates were prepared using RIPA lysis buffer (50 mM Tris-HCl, pH 8.0, 150 mM NaCl, 1% Triton X-100, 0.1% SDS, 0.5% sodium deoxycholate, supplemented with protease and phosphatase inhibitor cocktail tablets (Roche Diagnostics Corporation, Indianapolis, IN)). Proteins were resolved using standard SDS-PAGE, transferred to PVDF membrane (ThermoFisher Scientific, Waltham, MA) and probed with different antibodies. Pierce ECL Western Blotting substrate (ThermoFisher Scientific, Waltham, MA) was used for signal detection.

### RNA extraction, NanoString profiling, and quantitative real-time reverse-transcription PCR (qRT-PCR)

TRIzol reagent (Life Technologies, Grand Island, NY) was used to extract RNA from cell cultures and tumor tissues. miRNA Expression profiling was performed using nCounter miRNA expression assay (Nanostring® Technologies, Seattle, WA). TaqMan MicroRNA Reverse Transcription Kit and TaqMan MicroRNA assay kits (Applied Biosystems, Foster City, CA) were used for qRT-PCR determination of miRNA levels, performed on a 7300 Real-Time PCR System (Applied Biosystems, Foster City, CA). RNU6B RNA was used as the internal control to normalize sample input. Gene expression levels were determined using 2 ^−ΔΔCT^ method [45].

### Introduction of a *Dicer1*-expression construct into the *Dicer-Pten* DKO cancer cells and cell growth and adhesion analyses

A lentiviral FLAG-tagged *Dicer1*-expression construct EX-H0470-Lv101 was purchased from GeneCopoeia, Inc. (Rockville, MD). Lentiviral transduction particles were prepared by transfecting the lentiviral construct together with ViraPower™ Lentiviral Packaging Mix (ThermoFisher Scientific, Waltham, MA) into 293FT cells according to the manufacturer's recommendation. The expression of Dicer1 in transduced FTdT172 and FTdT967 tumor cells was confirmed by Western blot analysis using both a FLAG antibody (Cell Signaling Technology, Danvers, MA) and a Dicer1 antibody (Bethyl Laboratories, Montgomery, TX).

For BrdU incorporation and cell cycle analysis, cells were incubated with 10 μM of BrdU (Sigma-Aldrich, St. Louis, MO) for 1 h. After trypsinization, cells were fixed with 70% ethanol and chromosomal DNA was denatured using 2N HCl/0.5% Trion X-100 and neutralized using 0.1M Na_2_B_4_O_7_ (Sigma-Aldrich, St. Louis, MO). After resuspension, cells were labeled with FITC-conjugated anti-BrdU antibody (BD Biosciences, San Jose, CA) and 5 μg/mL of propidium iodide (Sigma-Aldrich, St. Louis, MO), and analyzed using a BD Accuri™ C6 cytometer (BD Biosciences, San Jose, CA).

Cell adhesion assay was performed using the Collagen I cell adhesion strips from Millicoat™ Screen kit ECM205 (EMD Millipore, Billerica, MA). 1×10^4^ cells were seeded to the strip wells and allowed to incubate for 1 hour. Nonadherent cells were washed away by phosphate buffered saline and the attached cells were stained using 0.2% crystal violet. The stain was solubilized in a 50:50 mixture of 0.1M sodium phosphate, pH 4.5 and 50% ethanol and read at 562 nm. For immunofluorescence, the mouse cells growing in an 8-well chamberslide (BD Biosciences, San Jose, CA) were fixed in 4% paraformaldehyde (Sigma-Aldrich, St. Louis, MO) and permeabilized with PBS containing 0.5% Triton X-100 (Sigma-Aldrich, St. Louis, MO). After blocking with 10% FBS, anti-ZO1 primary antibody (EMD Millipore, Billerica, MA) and Alexa Fluor 647-conjugated anti-mouse secondary antibody (Invitrogen, Carlsbad, CA) were added sequentially between washes. A mounting medium with DAPI (Vector Laboratories, Burlingame, CA) was used for counterstaining. Microscopic images were captured by a Leica DM IRE2 fluorescence microscope (Leica Microsystems, Bannockburn, IL) and analyzed by the OpenLab Cell Imaging System software (Leica Microsystems, Bannockburn, IL).

### Statistical analysis

All calculations were performed with MINITAB statistical software (Minitab, State College, PA). Significance of differences was determined using 2-tailed T-Test. A *P*-value of less than 0.05 was considered statistically significant for all tests.
